# Polysomnographic Sleep Dysregulation in Cocaine Dependence

**DOI:** 10.1100/tsw.2007.264

**Published:** 2007-11-02

**Authors:** Edwin M. Valladares, Michael R. Irwin

**Affiliations:** Cousins Center for Psychoneuroimmunology, University of California Los Angeles, Semel Institute for Neuroscience and Human Behavior, Los Angeles, USA

**Keywords:** cocaine, cocaine dependence, abstinence, sleep, REM sleep, slow wave sleep, polysomnography

## Abstract

Insomnia and sleep disturbance are associated with declines in health functioning, alongwith increases in mortality risk. Given the prominence of reported sleep disturbance incocaine-dependent subjects and persistence into recovery, understanding the natureand severity of these disturbances in this population may help to identify relevantpathways that contribute to the increased mortality in cocaine dependence. Polysomnography provides a means of objectively characterizing sleep and, in turn, sleep disturbances. Few studies have used polysomnography to evaluate sleep incocaine-dependent persons, yet these studies have the potential to advance treatmentsthat will ultimately reduce morbidity in cocaine-dependent subjects.

## INTRODUCTION

Insomnia and sleep disturbances have been associated with health declines in various diseased populations[1,2]. Among these are cocaine-dependent persons who complain of sleep disturbances[3,4] and are at a higher risk for stroke[5,6], cardiac infarction[7,8,9,10], and bacterial infection[11,12,13,14]. Although these sleep complaints have long been reported, relatively few studies have used polysomnographic sleep measures to assess sleep in this patient population[15,16,17,18,19,20,21].

Sleep behavior is coordinated through the central nervous system and can be measured via polysomnography (PSG). PSG is the process of acquiring and evaluating sleep objectively on the basis of electroencephalographic (EEG) recordings while the person sleeps. The simultaneous acquisition of other physiological signals aids in the identification and classification of sleep stages and sleep disorders (e.g., obstructive sleep apnea and periodic leg movements in sleep). Three physiological signals encompass a basic polysomnogram, which include four monopolar EEG sites ($C_3$-$A_2$, $C_4$-$A_1$, $O_1$-$A_2$, $O_2$-$A_1$), electroculogram, and submental electromyogram (EMG). The interested reader can find further technical information in one of the standard sleep medicine or PSG textbooks[22,23]. Once the polysomnogram is acquired, these data are evaluated by giving each 30 sec of sleep a score to identify the stage of sleep it represents. These stages include: 1, 2, 3, 4, rapid eye movement (REM), movement, and wake. Thus far, the Rechtschaffen and Kales (R & K) guidelines have been used as the standard for scoring practices[24]. All PSG sleep studies presented here have been scored using the R & K guidelines.

Once sleep is characterized by different stages, variables can be extracted to describe three domains: sleep continuity, sleep architecture, and REM measures. Sleep continuity measures quantify quality of sleep, which includes three variables: total sleep time (amount of sleep across the night), sleep efficiency ([total sleep time ÷ by time in bed] × 100), and sleep onset time (amount of time to sleep initiation). Sleep architecture provides a quantification of sleep minutes and/or percents in each stage of sleep. REM measures REM latency time (time to REM sleep from sleep onset time), and REM density or REM pressure (propensity for REM sleep). Taken together, these variables can provide objective sleep measures that can be used to describe sleep and sleep disturbances in virtually any diseased population.

The sleeping person moves through these stages repeatedly through the night. Healthy humans enter non-REM sleep from wakefulness. From non-REM sleep, there is a transition to REM sleep. After a period of REM sleep, there may or may not be a brief arousal or awakening. These progressions from wakefulness through stages of sleep repeat to form cycles. In healthy adults, there will be four to six NREM-REM cycles of about 80- to 110-min duration during the night.

The following is a mini-review of the studies that have characterized sleep polysomnographically in cocaine-dependent persons. Although most of these studies have used less than nine participants in each of their reports, a comparison will be made using their reported sleep continuity measures, sleep architecture, and REM measures.

## POLYSOMNOGRAPHIC SLEEP IN COCAINE DEPENDENCE – ACUTE ABSTINENCE

Different findings have emerged from the seven PSG studies reporting sleep in association with cocaine use. These differences may be attributed to the low number of participants, comorbidities, gender differences, and/or history of cocaine usage. For instance, five out of the seven studies reported their findings using less than nine cocaine-dependent persons[15,16,17,18,19,25], one of these studies considered cocaine-dependent individuals and methamphetamine users in the same cohort[17], and another examined the effects of cocaine administration in three recreational cocaine users[16]. Although different PSG findings have been reported, the overarching trend is that sleep continuity shows a deteriorated or fragmented pattern in cocaine-dependent persons, as described below. Furthermore, each study has improved on its predecessors' findings by using more stringent inclusion criteria, and increasing the number of participants.

From the six studies examining sleep during abstinence in cocaine dependence, five studies reported sleep continuity deterioration across abstinence. Sleep continuity deterioration included decreases in total sleep time[15,17,19,20], decreases in sleep efficiency[15,19], and increases in sleep onset time[15,18,19,20]. Decreases in total sleep time have been associated with declines in health functioning and increased risk of mortality[26].

Sleep architecture alterations were reported in all six studies. These sleep alterations included increases in REM sleep minutes or REM sleep percents of total sleep time[15,17], and loss of slow wave sleep (stages 3 and 4)[20,21]. Morgan and colleagues and Valladares et al. were among the first to report loss of slow wave sleep. It is an interesting finding, because both studies excluded participants with a history of head injury, which could have been a confounding factor[27]. This loss of slow wave sleep could be considered as premature aging, being that loss of slow wave sleep is noted in the elderly[28,29,30]. It is unclear why the previous four studies did not see this type of slow wave sleep loss. However, consideration should be given to the larger sample sizes in these two latter reports, with each reported findings on the basis of 12 and 17 cocaine-dependent participants, respectively, making them the two largest PSG studies to date.

Changes in REM measures have also been reported, which are consistent with the previously noted increases in REM sleep. Alterations in REM measures include decreases in REM latency time[15,18,21] and increases in density[15], which can be translated as increased propensity for REM sleep. REM sleep has been shown to be accompanied by increases in sympathetic output in healthy controls[31,32].

Although autonomic function during sleep has not been explored in cocaine-dependent populations, alcohol-dependent men also exhibit increases in sympathetic output during REM sleep[33,34]. Increases in sympathetic output are thought to contribute to the occurrence of cardiac arrhythmias and sudden cardiac death[35,36]. Furthermore, REM sleep predominates in the second half of the night, and the frequency of ventricular arrhythmias and sudden cardiac death, as well as other cardiac events such as stroke, increases twofold in the early morning or following awakening from sleep[37,38,39,40,41]. Taken together, the increases in REM sleep and decreases in REM latency might put cocaine-dependent persons at risk for cardiac arrhythmias and sudden cardiac death during acute abstinence.

## CONCLUSIONS

The studies presented here provide support for the hypothesis that sleep shows deterioration in measures of continuity and alterations in sleep architecture in cocaine-dependent persons. Given prior evidence that disturbances in sleep are associated with mortality risk, these data have implications for understanding the behavioral factors that contribute to morbid outcomes in cocaine dependence. Although the mechanisms that account for alterations of sleep in cocaine dependence are not known, these few studies provide insight into new directions in treatment. Moreover, these studies also uncover the necessity for additional PSG studies that would expand on these results by increasing the sample size, addressing the question of how age affects loss of slow wave sleep, and exploring the effects of prolonged abstinence on sleep.

## ACKNOWLEDGMENTS

This work was supported in part by National Institutes of Health Grants AA13239, DA16541, T32-MH19925, and M01 RR00827; the General Clinical Research Centers Program; and the Cousins Center for Psychoneuroimmunology. The authors thank the NIH, NIDA, and NIAAA for their support, and Sam Eljammal, M.D. and Anand Iyer for their efforts in the sleep laboratory.
